# Supplementary material for: Telehealth use among pediatric Alabama Medicaid enrollees, March-December 2020: Variations by race/ethnicity & place of residence
Source: PLoS One. 2023 Jun 26;18(6):e0287598. doi: 10.1371/journal.pone.0287598 (PMC10292692; doi:10.1371/journal.pone.0287598)
Supplement: S1 Appendix — (DOCX) [file pone.0287598.s001.docx]

**APPENDIX**

**Table A1: Baseline Variations in Any Telehealth Use by Race/Ethnicity, RUCA, Zip-Code Level Poverty & Broadband Access among Alabama Pediatric Medicaid Enrollees during 2018 & 2019^a^**

|  | Did Not Use Any Telehealth  N (%) | Used Any Telehealth  N (%) |
| --- | --- | --- |
|  |  |  |
| **Overall** | 1,288,774 (99.84) | 2,056 (0.16) |
| **Race/Ethnicity** |  |  |
| Non-Hispanic White | 453,096 (99.84) | 744 (0.16) |
| Non-Hispanic Black | 488,080 (99.82) | 888 (0.18) |
| Hispanic | 78,72 (99.95) | 40 (0.05) |
| Native American | 3,715(99.84) | 6 (0.16) |
| Asian | 9,962 (100.00) | 0 (0.00) |
| Other | 255,198 (99.85) | 0.15 |
| **RUCA Category^b^** |  |  |
| Urban | 962,686 (99.81) | 1,790 (0.19) |
| Large rural | 178,760 (99.89) | 189 (0.11) |
| Small rural | 96,698 (99.96) | 37 (0.04) |
| Isolated | 50,630 (99.92) | 40 (0.08) |
| **Zip-code Poverty Quartile** |  |  |
| Poverty Quartile 1 | 206,849 (99.89) | 235 (0.11) |
| Poverty Quartile 2 | 323,836 (99.77) | 744 (0.23) |
| Poverty Quartile 3 | 360,091 (99.88) | 432 (0.12) |
| Poverty Quartile 4 | 397,658 (99.84) | 645 (0.16) |
| **No Broadband Connectivity Decile** |  |  |
| First-ninth decile | 1,147,085 (99.83) | 1,941 (0.17) |
| Highest decile | 141,689 (99.92) | 115 (0.08) |
| **Biological Sex** |  |  |
| Male | 660,748 (99.82) | 1,218 (0.18) |
| Female | 628,026 (99.87) | 838 (0.13) |
| **Age Groups** |  |  |
| 0-3 years | 360,287 (100.00) | 18 (0.00) |
| 3-6 years | 201,959 (99.90) | 203 (0.10) |
| 6-12 years | 396,489 (99.76) | 947 (0.24) |
| 12-19 years | 330,039 (99.73) | 888 (0.27) |
| N=1,290,830; ^a^ Excluded January and February data for both years; **^b^** RUCA: Rural urban commuting area | | |
