# Supplementary material for: Telehealth use among pediatric Alabama Medicaid enrollees, March-December 2020: Variations by race/ethnicity & place of residence
Source: PLoS One. 2023 Jun 26;18(6):e0287598. doi: 10.1371/journal.pone.0287598 (PMC10292692; doi:10.1371/journal.pone.0287598)
Supplement: Attachment — Submitted filename: Response_to_Reviewer_Comments.docx [file pone.0287598.s002.docx]

PONE-D-22-31503

Telehealth Use Among Pediatric Alabama Medicaid Enrollees, March-December 2020: Variations by Race/Ethnicity & Place of Residence

Response to Editorial & Reviewer Comments.

**We are grateful for the thoughtful and positive reviews of our work and the suggestions that have helped us strengthen our work. We have addressed the issues raised and provided an item-by-item response to the comments from the editor and reviewer below.**

Journal Requirements:

When submitting your revision, we need you to address these additional requirements.

1. Please ensure that your manuscript meets PLOS ONE's style requirements, including those for file naming. The PLOS ONE style templates can be found at

<https://journals.plos.org/plosone/s/file?id=wjVg/PLOSOne_formatting_sample_main_body.pdf> and

[https://journals.plos.org/plosone/s/file?id=ba62/PLOSOne_formatting_sample_title_authors_affiliations.pdf](https://nam12.safelinks.protection.outlook.com/?url=https%3A%2F%2Fjournals.plos.org%2Fplosone%2Fs%2Ffile%3Fid%3Dba62%2FPLOSOne_formatting_sample_title_authors_affiliations.pdf&data=05%7C01%7Cbsen%40uab.edu%7Cb5586ce6595b46ec7bab08daf8a8b159%7Cd8999fe476af40b3b4351d8977abc08c%7C1%7C0%7C638095701640075855%7CUnknown%7CTWFpbGZsb3d8eyJWIjoiMC4wLjAwMDAiLCJQIjoiV2luMzIiLCJBTiI6Ik1haWwiLCJXVCI6Mn0%3D%7C3000%7C%7C%7C&sdata=fUw8XHtCHCG6VPRGd1dsBVd7H%2BRy2a1Vb05uPnBdXew%3D&reserved=0).

**Thank you. We have formatted the paper following PLOS ONE style requirements.**

2. Please provide additional details regarding participant consent. In the ethics statement in the Methods and online submission information, please ensure that you have specified (1) whether consent was informed and (2) what type you obtained (for instance, written or verbal, and if verbal, how it was documented and witnessed). If your study included minors, state whether you obtained consent from parents or guardians. If the need for consent was waived by the ethics committee, please include this information.

If you are reporting a retrospective study of medical records or archived samples, please ensure that you have discussed whether all data were fully anonymized before you accessed them and/or whether the IRB or ethics committee waived the requirement for informed consent. If patients provided informed written consent to have data from their medical records used in research, please include this information.

**We are reporting a retrospective study using administrative claims data for all pediatric enrollees in Alabama Medicaid. The data was obtained through a research contract funded by Alabama Medicaid Agency, was anonymized (though it included information on county and zipcode of residence) and is housed on secured data servers on our university campus, and can only be accessed by authorized members of the research team. Individual Medicaid enrollees were not approached for consent since that was logistically and financially infeasible (we had claims data for over 640,000 enrollees). The Institutional Review Board at the University of Alabama at Birmingham reviewed and approved this research.**

3. In your Data Availability statement, you have not specified where the minimal data set underlying the results described in your manuscript can be found. PLOS defines a study's minimal data set as the underlying data used to reach the conclusions drawn in the manuscript and any additional data required to replicate the reported study findings in their entirety. All PLOS journals require that the minimal data set be made fully available. For more information about our data policy, please see <http://journals.plos.org/plosone/s/data-availability>.

Upon re-submitting your revised manuscript, please upload your study’s minimal underlying data set as either Supporting Information files or to a stable, public repository and include the relevant URLs, DOIs, or accession numbers within your revised cover letter. For a list of acceptable repositories, please see <http://journals.plos.org/plosone/s/data-availability#loc-recommended-repositories>. Any potentially identifying patient information must be fully anonymized.

**This is a third-party dataset that belongs to Alabama Medicaid, which we are given access to via a research contract with Alabama Medicaid. Thus, we are unable to make a minimal dataset available through a data repository, but interested researchers can contact Alabama Medicaid Agency (**[**https://medicaid.alabama.gov/)**](https://medicaid.alabama.gov/)) **to access it. We note that several other papers published in the PLOS journals that use Medicaid data have had similar restrictions on data sharing (**[**https://journals.plos.org/plosone/article?id=10.1371/journal.pone.0122927**](https://journals.plos.org/plosone/article?id=10.1371/journal.pone.0122927)**,** [**https://journals.plos.org/plosone/article?id=10.1371/journal.pone.0144965**](https://journals.plos.org/plosone/article?id=10.1371/journal.pone.0144965)**,** [**https://journals.plos.org/plosone/article?id=10.1371/journal.pone.0248360**](https://journals.plos.org/plosone/article?id=10.1371/journal.pone.0248360)**,**

[**https://journals.plos.org/plosone/article?id=10.1371/journal.pone.0241298**](https://journals.plos.org/plosone/article?id=10.1371/journal.pone.0241298)**)**

Important: If there are ethical or legal restrictions to sharing your data publicly, please explain these restrictions in detail. Please see our guidelines for more information on what we consider unacceptable restrictions to publicly sharing data: <http://journals.plos.org/plosone/s/data-availability#loc-unacceptable-data-access-restrictions>. Note that it is not acceptable for the authors to be the sole named individuals responsible for ensuring data access.

We will update your Data Availability statement to reflect the information you provide in your cover letter.

4. We note that you have stated that you will provide repository information for your data at acceptance. Should your manuscript be accepted for publication, we will hold it until you provide the relevant accession numbers or DOIs necessary to access your data. If you wish to make changes to your Data Availability statement, please describe these changes in your cover letter, and we will update your Data Availability statement to reflect the information you provide.

**After a discussion with Alabama Medicaid, we would like to change our Data Availability statement to the following:**

**“This research was conducted using administrative claims data from Alabama Medicaid, which we accessed through a research contract with Alabama Medicaid. We are prohibited from publicly making this data available. However, interested researchers can contact us for the data after obtaining written permission from the Alabama Medicaid Agency to access the data (**[**https://medicaid.alabama.gov/)**](https://medicaid.alabama.gov/))**”.**

[Note: HTML markup is below. Please do not edit.]

Reviewers' comments:

Reviewer's Responses to Questions

**Comments to the Author**

1. Is the manuscript technically sound, and do the data support the conclusions?

The manuscript must describe a technically sound piece of scientific research with data that supports the conclusions. Experiments must have been conducted rigorously, with appropriate controls, replication, and sample sizes. The conclusions must be drawn appropriately based on the data presented.

Reviewer #1: Yes

2. Has the statistical analysis been performed appropriately and rigorously?

Reviewer #1: Yes

3. Have the authors made all data underlying the findings in their manuscript fully available?

The [PLOS Data policy](https://nam12.safelinks.protection.outlook.com/?url=http%3A%2F%2Fwww.plosone.org%2Fstatic%2Fpolicies.action%23sharing&data=05%7C01%7Cbsen%40uab.edu%7Cb5586ce6595b46ec7bab08daf8a8b159%7Cd8999fe476af40b3b4351d8977abc08c%7C1%7C0%7C638095701640232085%7CUnknown%7CTWFpbGZsb3d8eyJWIjoiMC4wLjAwMDAiLCJQIjoiV2luMzIiLCJBTiI6Ik1haWwiLCJXVCI6Mn0%3D%7C3000%7C%7C%7C&sdata=Poy3RuznbhhH1EHjip5NDqwcgtXube6iiqGfp5Sx8H8%3D&reserved=0) requires authors to make all data underlying the findings described in their manuscript fully available without restriction, with rare exception (please refer to the Data Availability Statement in the manuscript PDF file). The data should be provided as part of the manuscript or its supporting information, or deposited to a public repository. For example, in addition to summary statistics, the data points behind means, medians and variance measures should be available. If there are restrictions on publicly sharing data—e.g. participant privacy or use of data from a third party—those must be specified.

Reviewer #1: Yes

4. Is the manuscript presented in an intelligible fashion and written in standard English?

PLOS ONE does not copyedit accepted manuscripts, so the language in submitted articles must be clear, correct, and unambiguous. Any typographical or grammatical errors should be corrected at revision, so please note any specific errors here.

Reviewer #1: Yes

5. Review Comments to the Author

Please use the space provided to explain your answers to the questions above. You may also include additional comments for the author, including concerns about dual publication, research ethics, or publication ethics. (Please upload your review as an attachment if it exceeds 20,000 characters)

Reviewer #1: This is a nice paper with new results. It has a large sample and robust methods. Writing is also good.

Here are areas for improvement (Most of them about nuances):

1- Genders differ in all processes related to health care use. In this paper, however, gender differences in correlations are not shown. Only main effects of gender are shown. This is like gender is a confounder or a covariate with only main effects. Some of the effects of gender are indirect through changing pathways.

**Thank you for your positive comments on your paper and for your helpful suggestions. We agree that gender may change pathways of associations between race and place with telehealth use; hence, we now also present results that are stratified by gender in table 2.**

2- Age groups differ in correlates of health care use in part because differences in their health literacy and chronic disease. Thus, show us if older people and older-old people differ in these paths.

**Thank you for your comment. We agree that age may change pathways of associations between race and place with telehealth use. Since we focus on the pediatric population (<19 years), the distinction between older and older-old is not directly relevant, but we believe differences may exist between pre-teen and teen children since the latter may have more of a “say” in their own health utilization decisions. Hence, we stratify results by <12 years and 12-<19 years.**

3- Race and ethnic groups differ in correlates of health care use. This means race and ethnicity are not just confounders but also moderators of the SES effects.

Based on minorities' diminished returns, SES has stronger effects on health and health care use of Whites than men. That is, due to racism and social stratification, SES loses some of its effect for marginalized people particularly Black people.
Some examples are here:
<https://pubmed.ncbi.nlm.nih.gov/32783022/>
<https://pubmed.ncbi.nlm.nih.gov/32457934/>
<https://pubmed.ncbi.nlm.nih.gov/32190811/>

**Thank you for your comment. To be clear, we do not treat race and ethnicity as confounders. Rather, they are the main exposure variables of interest, and we are interested in exploring racial and ethnic disparities (as well as disparities by place of residence) in telehealth utilization. That said, future research should explore the interplay of race/ethnicity and place in various telehealth service utilization, and we now mention this in the Limitations and state that this is an area for future research.**

**Also, thank you for making suggestions regarding additional citations. Since they pertained to the broader adult population while our study focuses on the publicly-insured pediatric population, not all of them were very directly relevant. However, we did cite** [**https://pubmed.ncbi.nlm.nih.gov/32457934/**](https://pubmed.ncbi.nlm.nih.gov/32457934/) **in the introduction as one example of disparities by race across education levels in the existing literature.**

4- This paper only focuses on patient data. Neighborhood, health care system, health care provider, and geographic distribution of resources are not even considered.

**We agree that neighborhood, health care system, health care provider, and geographic distribution of resources are important, and indeed we accounted for several of those in our model. Specifically, we included zip-code level poverty quartiles, zip-code level broadband access (especially relevant for telehealth), and family medicine or pediatric physicians per 1000 population in the county. Further, in response to the reviewer’s comment about healthcare systems, we also included hospital beds per 1000 in the models. These additional variables are listed clearly in the notes below in Tables 1, 2, and 3 as model control variables.**

After these issues are addressed, I will be happy to recommend acceptance.

**Thank you. We believe we have been able to address the issues that you raised and thank you for your thoughtful comments.**
