# Supplementary material for: Telehealth use among pediatric Alabama Medicaid enrollees, March-December 2020: Variations by race/ethnicity & place of residence
Source: PLoS One. 2023 Jun 26;18(6):e0287598. doi: 10.1371/journal.pone.0287598 (PMC10292692; doi:10.1371/journal.pone.0287598)
Supplement: Attachment — Submitted filename: Response to Reviewer_Plos_One_april23.docx [file pone.0287598.s003.docx]

We are grateful to the reviewer for the detailed and valuable feedback provided. We are also grateful that the reviewer stated that we adequately addressed all points raised in the prior review.

Below, we outline specific changes we made, with reviewer comments in plain text followed by our response in bold text.

Major concerns:
1. The measurements of the variables are not clearly described. The authors only talked about what the variables were included, but did not explain how they were categorized or applied.
a. For example, the patients mentioned the RUCA codes was used to categorize the residence areas but did not elaborate how the specific codes were applied. They also did not mention the version of the RUCA codes, and there is no reference for the RUCA code, which could be confusing for the readers who are not familiar with this field.

**Thank you for this comment. We have used the latest RUCA codes, revised and released in July 2019, and provided details about RUCA in the methods section, including the citation for the most recent version of RUCA codes (‘Materials and Methods’ subsection, para 2).**

b. Similarly, what’s the rationale of using quartiles of zip-code level poverty. Is there any reference?

**We have included citations that support using zip-code level poverty quartiles as a covariate in our model (‘Materials and Methods’ subsection, para 2). Examples include:**

<https://www.sciencedirect.com/science/article/pii/S0022480415003510>

<https://acsjournals.onlinelibrary.wiley.com/doi/10.1002/cncr.21732>

<https://journals.sagepub.com/doi/10.1177/107327480901600210>

2. There is no data and baseline information on the patients, especially the information on confounders is missing.

**We agree that this would be useful information for the reader. We have included the baseline information on variation in telehealth utilization by the enrollee characteristics during comparable periods in 2018 & 2019 in the Appendix. Note that this also helps address the reviewer’s later question about telehealth use before the pandemic.**

3. Is it possible to conduct a Poisson mixed effects model, as there might be fixed differences between residence areas but homogeneity within residence areas?

**In response to this suggestion, we have changed our model to Poisson mixed effects model.**

4. Is it possible to consider the impact of the time, as the usage of telehealth in March and in December could be different?

**The number of months an enrollee was in the program is used as an exposure variable in the model. Since our research question is not focused on monthly trends per se, we did not report on monthly rates of telehealth use in this paper, but we have cited a CMS source that reports monthly trends in Medicaid use in telehealth for the country as a whole (**[**https://www.medicaid.gov/state-resource-center/downloads/covid19-data-snapshot-11122021.pdf**](https://www.medicaid.gov/state-resource-center/downloads/covid19-data-snapshot-11122021.pdf)**)**

Minor comments:
1. Is there a specific reason why the study period was only in 2020?

**We selected this study period to understand the initial impact of the pandemic on telehealth utilization among pediatric enrollees of the Alabama Medicaid program. There is a substantial lag in time before we receive the completed Alabama Medicaid claims data for any calendar year; hence using data for the latter part of the pandemic was not feasible for this manuscript, though we hope to investigate that in future research.**

2. What was the telehealth use rate before the pandemic? Is there any published literature about this?

**As mentioned earlier, we have now reported telehealth use among Alabama’s pediatric Medicaid population in 2018 and 2019 in the appendix. To the best of our knowledge, there is no published literature about the telehealth utilization rate of pediatric enrollees in the Alabama Medicaid program. However, we have provided citations that provide information on the telehealth utilization trends among all Medicaid enrollees.**
[**https://www.ncbi.nlm.nih.gov/pmc/articles/PMC9290332/**](https://www.ncbi.nlm.nih.gov/pmc/articles/PMC9290332/)

3. Enrollees in Pov_Q3 were more likely to use telehealth, while those in Pov_Q4 were less likely. What’s the potential reason for this difference?

**In the updated mixed effects models, we do not find any statistically significant association between poverty quartiles and telehealth utilization. Thus, we have updated the Results and Discussion sections to reflect this.**

4. Tables: Does “Other” of Race/Ethnicity means both other and unknown?

**Thank you for this question. We have now clarified that” Other” includes both “other and unknown” and the method section of the manuscript.**
